# Bis(9-amino­acridinium) bis­(pyridine-2,6-dicarboxyl­ato)cuprate(II) trihydrate

**DOI:** 10.1107/S1600536810037499

**Published:** 2010-09-30

**Authors:** Hossein Aghabozorg, Shabnam Ahmadvand, Masoud Mirzaei, Hamid Reza Khavasi

**Affiliations:** a Faculty of Chemistry, Islamic Azad University, North Tehran Branch, Tehran, Iran; b Department of Chemistry, School of Sciences, Ferdowsi University of Mashhad, Mashhad, Iran; c Departement of Chemistry, Shahid Beheshti University, G. C., Evin, Tehran 1983963113, Iran

## Abstract

The asymmetric unit of the title compound, (C_13_H_11_N_2_)_2_[Cu(C_7_H_3_NO_4_)_2_]·3H_2_O or (9-aminoAcr)[Cu(pydc)_2_]·3H_2_O, contains a Cu(pydc)_2_ (pydc = pyridine-2,6-dicarboxyl­ate) anion, two protonated 9-amino­acridine (9-aminoAcr)^+^ counter-ions and three uncoordinated water mol­ecules. The anion contains a six-coordinated Cu(II) atom within a distorted octa­hedral geometry. Non-covalent inter­actions *i.e.* N—H⋯O and O—H⋯O hydrogen bonds and inter­molecular π–π contacts between the pyridine rings [centroid–centroid distance = 3.7773 (13) Å] and acridine rings [centroid–centroid distance = 3.4897 (13), 3.7784 (14) and 3.8627 (15) Å] result in the formation of a three-dimensional network.

## Related literature

For related structures, see: Aghabozorg *et al.* (2008 ▶, 2010 ▶); Eshtiagh-Hosseini *et al.* (2010 ▶); Tabatabaee *et al.* (2009 ▶). An independent determination of the title compound is reported in the preceeding paper by Derikvand *et al.* (2010 ▶).
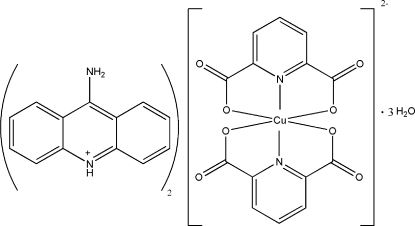


## Experimental

### 

#### Crystal data

(C_13_H_11_N_2_)_2_[Cu(C_7_H_3_NO_4_)_2_]·3H_2_O
*M* *_r_* = 838.27
Triclinic, 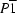

*a* = 10.921 (2) Å
*b* = 13.299 (3) Å
*c* = 14.008 (3) Å
α = 102.09 (3)°
β = 103.96 (3)°
γ = 105.38 (3)°
*V* = 1820.6 (9) Å^3^
*Z* = 2
Mo *K*α radiation
μ = 0.67 mm^−1^
*T* = 298 K
0.38 × 0.30 × 0.25 mm

#### Data collection

STOE IPDS II diffractometer
Absorption correction: numerical [shape of crystal determined optically (*X-RED32* and *X-SHAPE*; Stoe & Cie, 2005 ▶)] *T*_min_ = 0.787, *T*_max_ = 0.849
21686 measured reflections
9767 independent reflections
7989 reflections with *I* > 2σ(*I*)
*R*_int_ = 0.027

#### Refinement

*R*[*F* ^2^ > 2σ(*F* ^2^)] = 0.038
*wR*(*F* ^2^) = 0.094
*S* = 1.03
9767 reflections
571 parameters
H atoms treated by a mixture of independent and constrained refinement
Δρ_max_ = 0.25 e Å^−3^
Δρ_min_ = −0.38 e Å^−3^

### 

Data collection: *X-AREA* (Stoe & Cie, 2005 ▶); cell refinement: *X-AREA*; data reduction: *X-AREA*; program(s) used to solve structure: *SHELXS97* (Sheldrick, 2008 ▶); program(s) used to refine structure: *SHELXL97* (Sheldrick, 2008 ▶); molecular graphics: *ORTEP-3 for Windows* (Farrugia, 1997 ▶); software used to prepare material for publication: *WinGX* (Farrugia, 1999 ▶).

## Supplementary Material

Crystal structure: contains datablocks I. DOI: 10.1107/S1600536810037499/vm2039sup1.cif

Structure factors: contains datablocks I. DOI: 10.1107/S1600536810037499/vm2039Isup2.hkl

Additional supplementary materials:  crystallographic information; 3D view; checkCIF report

## Figures and Tables

**Table 1:** Hydrogen-bond geometry (Å, °)

*D*—H⋯*A*	*D*—H	H⋯*A*	*D*⋯*A*	*D*—H⋯*A*
O1*W*—H1*WA*⋯O7	0.87 (4)	1.98 (4)	2.832 (2)	167 (4)
O1*W*—H1*WB*⋯O4^i^	0.74 (4)	2.14 (4)	2.877 (3)	175 (4)
O2*W*—H2*WA*⋯O8^ii^	0.84 (3)	1.98 (3)	2.820 (3)	174 (3)
O2*W*—H2*WB*⋯O8	0.84 (4)	1.98 (4)	2.810 (3)	174 (4)
O3*W*—H3*WA*⋯O2^iii^	0.79 (3)	2.01 (3)	2.787 (3)	169 (3)
O3*W*—H3*WB*⋯O2*W*^iv^	0.84 (3)	1.89 (3)	2.730 (3)	175 (3)
N3—H3*B*⋯O6	0.83 (2)	1.88 (2)	2.716 (2)	179 (3)
N4—H4*B*⋯O1*W*^v^	0.89 (3)	2.09 (3)	2.938 (3)	158 (2)
N4—H4*C*⋯O8^v^	0.77 (3)	2.27 (3)	2.972 (2)	152 (3)
N5—H5*B*⋯O3*W*	0.80 (2)	1.91 (2)	2.703 (2)	173 (2)
N6—H6*A*⋯O2^vi^	0.87 (2)	1.97 (2)	2.819 (2)	164 (2)
N6—H6*B*⋯O5	0.83 (3)	2.11 (3)	2.888 (2)	158 (2)

Symmetry codes: (i) 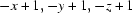; (ii) 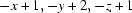; (iii) 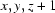; (iv) 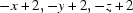; (v) 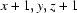; (vi) 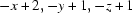.

**Fig. 1.**
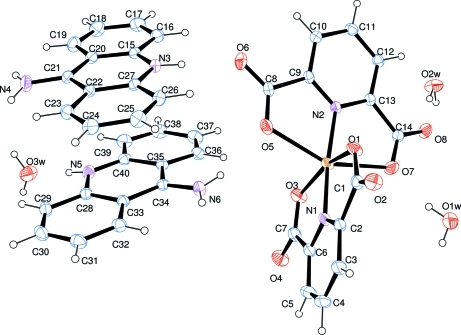
The molecular structure of the title molecule, with the atom-numbering scheme. Displacement ellipsoids are drawn at the 50% probability level.

**Fig. 2.**
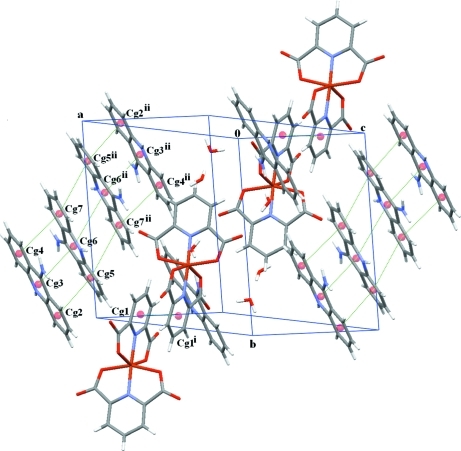
Extensive π···π stacking interaction between aromatic rings of 9-aminoAcr ions, (9-aminoAcr)+, with centroid-centroid distances ranging from 3.4897 (13)–3.8627 (15) Å. Cg denotes the ring centroid; Cg1=N2/C9-C13, Cg2=C15-C20, Cg3=N3/C15/C20-C22/C27, Cg4=C22-C27, Cg5=C35-C40, Cg6=N5/C28/C33-C35/C40, Cg7=C28-C33. [Symmetry codes : (i) 2-x, 2-y, 1-z; (ii) 2-x, 1-y, 2-z]

## supplementary crystallographic information

### Comment

PydcH_2_ as achelating ligand with steric hindrance and weak stacking interactions can offer possibilities to form coordination polymers or dimers through carboxylate bridging. It is known that this ligand exhibits various coordination modes with possible monodentate, bidentate, tridentate, or bridging in transitionmetal-pydc complexes, depending on the prescence of whether the divalent anionic (pydc)^2-^, protonated anionic (pydcH)^-^ or fully protonated (pydcH_2_). In recent years, our research group has been interested in the synthesis of proton transfer compounds including different dicarboxylic acid especially pydcH_2 _bearing a number of organic donor ligands containing N, S, and O atoms and in the study of their behavior with diverse metal ions. In this regard, we have reported some proton transfer compounds with acridine (Tabatabaee *et al.*, 2009, Eshtiagh-Hosseini *et al.*, 2010) and proton transfer compounds with various donor and acceptor fragments (further details and related literature see Aghabozorg *et al.*, 2008). We describe here the crystal structure of a new coordination compound based upon Cu^II^ atom, pydcH_2_, and 9-aminoAcr. The title compound contains a [Cu(pydc)_2_]^2-^ anion, two (9-aminoAcr)^+^ and three uncoordinated water molecules (Fig. 1). In the anion fragment, the Cu^II^ atom is six-coordinated by two N atoms (N1 and N2) and four O atoms (O1, O3, O5 and O7) from the carboxylate groups of two (pydc)^2-^ ligands, with bond length ranges of 1.9128 (15)–2.3509 (16) Å. The N1—Cu1—N2 [174.31 (6)°], O1—Cu1—O3 [159.47 (5)°] and O5—Cu1—O7 [151.61 (5)°] angles show that the four carboxylate groups of the two (pydc)^2-^ ligands orient in a flattened tetrahedral arrangement around the central atom. The coordination environment around Cu^II^ is distorted octahedral.

In the crystal structure, intermolecular O—H···O and N—H···O hydrogen bonds (Table 1) and π···π contacts between pyridine rings *Cg*1···*Cg*1^i^ [symmetry code: (i) 2 - *x*, 2 - *y*,1 - *z*, where *Cg*1 is the centroid of ring N2/C9—C13] and between adjacent acridine rings with centroid-centroid distances between 3.4897 (13) and 3.8627 (15) Å (Fig. 2) stabilize the structure. Furthermore water molecules acting as good gluing factors increase the stability of the crystalline network; further details and related literature about water clusters has been presented in a review article (Aghabozorg *et al.*, 2010).

### Experimental

The reaction of copper^II^ nitrate trihydrate (241 mg, 1 mmol), 9-aminoAcr (389 mg, 2 mmol) and pydcH_2_ (334 mg, 2 mmol) in a 1:2:2 molar ratio in aqueous/ethanolic solution resulted in the formation of green block (9-aminoAcr)^+^_2_.[Cu(pydc)_2_].3H_2_O crystals.

### Refinement

C-bound H-atoms were positioned geometrically, with C—H=0.93Å and constrained to ride on their parent atoms with *U*_iso_(H)=1.2*U*_eq_. N-H and O-H (water) H-atoms were located in a difference Fourier map and then refined isotropically.

### Crystal data

**Table:**

(C_13_H_11_N_2_)_2_[Cu(C_7_H_3_NO_4_)_2_]·3H_2_O	*Z* = 2
*M**_r_* = 838.27	*F*(000) = 866
Triclinic, *P*1	*D*_x_ = 1.529 Mg m^−^^3^
Hall symbol: -P 1	Mo *K*α radiation, λ = 0.71073 Å
*a* = 10.921 (2) Å	Cell parameters from 342 reflections
*b* = 13.299 (3) Å	θ = 1.7–29.3°
*c* = 14.008 (3) Å	µ = 0.67 mm^−^^1^
α = 102.09 (3)°	*T* = 298 K
β = 103.96 (3)°	Block, green
γ = 105.38 (3)°	0.38 × 0.30 × 0.25 mm
*V* = 1820.6 (9) Å^3^	

### Data collection

**Table:**

STOE IPDS II diffractometer	9767 independent reflections
Radiation source: fine-focus sealed tube	7989 reflections with *I* > 2σ(*I*)
graphite	*R*_int_ = 0.027
Detector resolution: 0.15 mm pixels mm^-1^	θ_max_ = 29.3°, θ_min_ = 1.7°
rotation method scans	*h* = −14→14
Absorption correction: numerical [shape of crystal determined optically (*X-RED32* and *X-SHAPE*; Stoe & Cie, 2005)]	*k* = −18→17
*T*_min_ = 0.787, *T*_max_ = 0.849	*l* = −19→19
21686 measured reflections	

### Refinement

**Table:**

Refinement on *F*^2^	Primary atom site location: structure-invariant direct methods
Least-squares matrix: full	Secondary atom site location: difference Fourier map
*R*[*F*^2^ > 2σ(*F*^2^)] = 0.038	Hydrogen site location: inferred from neighbouring sites
*wR*(*F*^2^) = 0.094	H atoms treated by a mixture of independent and constrained refinement
*S* = 1.03	*w* = 1/[σ^2^(*F*_o_^2^) + (0.0426*P*)^2^ + 0.6394*P*] where *P* = (*F*_o_^2^ + 2*F*_c_^2^)/3
9767 reflections	(Δ/σ)_max_ = 0.009
571 parameters	Δρ_max_ = 0.25 e Å^−^^3^
0 restraints	Δρ_min_ = −0.38 e Å^−^^3^

### Special details

**Table:**

Geometry. All e.s.d.'s (except the e.s.d. in the dihedral angle between two l.s. planes) are estimated using the full covariance matrix. The cell e.s.d.'s are taken into account individually in the estimation of e.s.d.'s in distances, angles and torsion angles; correlations between e.s.d.'s in cell parameters are only used when they are defined by crystal symmetry. An approximate (isotropic) treatment of cell e.s.d.'s is used for estimating e.s.d.'s involving l.s. planes.
Refinement. Refinement of *F*^2^ against ALL reflections. The weighted *R*-factor *wR* and goodness of fit *S* are based on *F*^2^, conventional *R*-factors *R* are based on *F*, with *F* set to zero for negative *F*^2^. The threshold expression of *F*^2^ > σ(*F*^2^) is used only for calculating *R*-factors(gt) *etc*. and is not relevant to the choice of reflections for refinement. *R*-factors based on *F*^2^ are statistically about twice as large as those based on *F*, and *R*- factors based on ALL data will be even larger.

### Fractional atomic coordinates and isotropic or equivalent isotropic displacement parameters (Å^2^)

**Table:**

	*x*	*y*	*z*	*U*_iso_*/*U*_eq_	
C1	0.92667 (19)	0.60711 (13)	0.42626 (12)	0.0349 (3)	
C2	0.82734 (19)	0.52885 (13)	0.45838 (12)	0.0336 (3)	
C3	0.7771 (2)	0.41642 (14)	0.42011 (14)	0.0434 (4)	
H3	0.8050	0.3797	0.3699	0.052*	
C4	0.6839 (2)	0.36009 (15)	0.45865 (16)	0.0518 (5)	
H4	0.6483	0.2844	0.4338	0.062*	
C5	0.6433 (2)	0.41524 (15)	0.53371 (16)	0.0479 (5)	
H5	0.5793	0.3777	0.5586	0.057*	
C6	0.70022 (19)	0.52775 (14)	0.57088 (13)	0.0365 (4)	
C7	0.6773 (2)	0.60503 (15)	0.65600 (14)	0.0405 (4)	
C8	1.12179 (19)	0.88335 (15)	0.73752 (13)	0.0374 (4)	
C9	1.05336 (17)	0.95035 (13)	0.68217 (12)	0.0300 (3)	
C10	1.11455 (18)	1.06105 (14)	0.69997 (13)	0.0359 (4)	
H10	1.2007	1.0969	0.7456	0.043*	
C11	1.04499 (19)	1.11726 (13)	0.64861 (14)	0.0375 (4)	
H11	1.0844	1.1914	0.6585	0.045*	
C12	0.91678 (18)	1.06222 (13)	0.58267 (13)	0.0333 (3)	
H12	0.8681	1.0990	0.5485	0.040*	
C13	0.86124 (17)	0.95140 (12)	0.56785 (12)	0.0289 (3)	
C14	0.72275 (18)	0.88280 (14)	0.49429 (13)	0.0347 (3)	
C15	1.37185 (16)	0.95961 (13)	1.07213 (13)	0.0324 (3)	
C16	1.32913 (19)	1.05136 (15)	1.07799 (16)	0.0418 (4)	
H16	1.2923	1.0673	1.0182	0.050*	
C17	1.3422 (2)	1.11666 (16)	1.17189 (18)	0.0495 (5)	
H17	1.3137	1.1769	1.1756	0.059*	
C18	1.3977 (2)	1.09398 (18)	1.26218 (17)	0.0540 (5)	
H18	1.4055	1.1389	1.3255	0.065*	
C19	1.4404 (2)	1.00637 (17)	1.25805 (15)	0.0478 (5)	
H19	1.4777	0.9924	1.3188	0.057*	
C20	1.42907 (17)	0.93619 (14)	1.16281 (13)	0.0356 (3)	
C21	1.46778 (17)	0.84053 (14)	1.15464 (13)	0.0341 (3)	
C22	1.45007 (16)	0.77425 (13)	1.05444 (13)	0.0312 (3)	
C23	1.48653 (18)	0.67858 (15)	1.03790 (15)	0.0387 (4)	
H23	1.5244	0.6573	1.0939	0.046*	
C24	1.4666 (2)	0.61789 (16)	0.94107 (16)	0.0437 (4)	
H24	1.4893	0.5547	0.9313	0.052*	
C25	1.4119 (2)	0.64989 (16)	0.85545 (15)	0.0441 (4)	
H25	1.4005	0.6084	0.7896	0.053*	
C26	1.37570 (19)	0.74056 (15)	0.86782 (14)	0.0386 (4)	
H26	1.3390	0.7607	0.8107	0.046*	
C27	1.39373 (16)	0.80415 (13)	0.96740 (13)	0.0308 (3)	
C28	1.17771 (16)	0.58124 (14)	1.05864 (13)	0.0338 (3)	
C29	1.23228 (18)	0.51486 (17)	1.10920 (16)	0.0436 (4)	
H29	1.2597	0.5332	1.1806	0.052*	
C30	1.24485 (19)	0.42350 (17)	1.05313 (18)	0.0492 (5)	
H30	1.2795	0.3789	1.0866	0.059*	
C31	1.2062 (2)	0.39600 (16)	0.94593 (19)	0.0476 (5)	
H31	1.2161	0.3336	0.9087	0.057*	
C32	1.15397 (18)	0.46015 (14)	0.89518 (15)	0.0384 (4)	
H32	1.1294	0.4415	0.8238	0.046*	
C33	1.13709 (16)	0.55450 (13)	0.95054 (13)	0.0308 (3)	
C34	1.08234 (16)	0.62463 (12)	0.90149 (12)	0.0288 (3)	
C35	1.06314 (16)	0.71546 (13)	0.96518 (12)	0.0302 (3)	
C36	0.99460 (19)	0.78033 (14)	0.92334 (14)	0.0368 (4)	
H36	0.9628	0.7663	0.8524	0.044*	
C37	0.9746 (2)	0.86353 (15)	0.98634 (17)	0.0467 (5)	
H37	0.9281	0.9052	0.9581	0.056*	
C38	1.0236 (2)	0.88633 (16)	1.09302 (17)	0.0517 (5)	
H38	1.0117	0.9447	1.1351	0.062*	
C39	1.0883 (2)	0.82497 (16)	1.13650 (15)	0.0455 (4)	
H39	1.1200	0.8409	1.2077	0.055*	
C40	1.10684 (17)	0.73667 (14)	1.07256 (13)	0.0339 (3)	
N1	0.78860 (15)	0.57997 (11)	0.53151 (10)	0.0326 (3)	
N2	0.93015 (14)	0.89696 (10)	0.61670 (10)	0.0279 (3)	
N3	1.35654 (15)	0.89429 (12)	0.97868 (11)	0.0333 (3)	
H3B	1.318 (2)	0.9069 (17)	0.9258 (18)	0.042 (6)*	
N4	1.51724 (19)	0.81276 (16)	1.23737 (14)	0.0467 (4)	
H4B	1.528 (2)	0.748 (2)	1.2318 (19)	0.057 (7)*	
H4C	1.528 (3)	0.850 (2)	1.291 (2)	0.067 (8)*	
N5	1.16460 (15)	0.67181 (13)	1.11570 (12)	0.0373 (3)	
H5B	1.176 (2)	0.6785 (19)	1.1757 (19)	0.048 (6)*	
N6	1.04770 (17)	0.60697 (13)	0.80144 (12)	0.0378 (3)	
H6A	1.054 (2)	0.5511 (19)	0.7603 (17)	0.044 (6)*	
H6B	1.030 (2)	0.654 (2)	0.7758 (19)	0.054 (7)*	
O1	0.95524 (15)	0.70686 (9)	0.47217 (10)	0.0418 (3)	
O2	0.97283 (16)	0.57032 (11)	0.35961 (11)	0.0493 (3)	
O3	0.74543 (16)	0.70499 (11)	0.67476 (11)	0.0478 (3)	
O4	0.59948 (17)	0.56796 (13)	0.70047 (12)	0.0566 (4)	
O5	1.06120 (15)	0.78245 (10)	0.70856 (10)	0.0442 (3)	
O6	1.22946 (16)	0.93578 (13)	0.80681 (12)	0.0601 (4)	
O7	0.69467 (15)	0.78226 (10)	0.47402 (11)	0.0497 (3)	
O8	0.64709 (14)	0.93215 (11)	0.45968 (11)	0.0454 (3)	
O1W	0.5812 (2)	0.62222 (17)	0.27969 (14)	0.0649 (5)	
H1WA	0.618 (3)	0.663 (3)	0.343 (3)	0.082 (10)*	
H1WB	0.538 (3)	0.574 (3)	0.288 (3)	0.085 (12)*	
O2W	0.61462 (19)	1.13490 (14)	0.52765 (15)	0.0605 (4)	
H2WA	0.536 (3)	1.119 (2)	0.531 (2)	0.069 (9)*	
H2WB	0.627 (3)	1.075 (3)	0.512 (3)	0.091 (11)*	
O3W	1.1854 (2)	0.70163 (17)	1.31659 (12)	0.0679 (5)	
H3WA	1.132 (3)	0.668 (2)	1.337 (2)	0.063 (8)*	
H3WB	1.248 (3)	0.749 (2)	1.366 (2)	0.066 (8)*	
Cu1	0.85947 (2)	0.735741 (15)	0.580449 (16)	0.03269 (6)	

### Atomic displacement parameters (Å^2^)

**Table:**

	*U*^11^	*U*^22^	*U*^33^	*U*^12^	*U*^13^	*U*^23^
C1	0.0476 (10)	0.0303 (7)	0.0268 (7)	0.0145 (7)	0.0114 (7)	0.0070 (6)
C2	0.0465 (10)	0.0275 (7)	0.0262 (7)	0.0145 (7)	0.0087 (7)	0.0069 (6)
C3	0.0631 (12)	0.0274 (8)	0.0353 (9)	0.0142 (8)	0.0131 (9)	0.0040 (7)
C4	0.0692 (14)	0.0252 (8)	0.0489 (11)	0.0057 (8)	0.0124 (10)	0.0064 (7)
C5	0.0574 (12)	0.0342 (9)	0.0487 (11)	0.0061 (8)	0.0179 (10)	0.0156 (8)
C6	0.0449 (10)	0.0332 (8)	0.0330 (8)	0.0125 (7)	0.0117 (7)	0.0144 (7)
C7	0.0496 (11)	0.0411 (9)	0.0363 (9)	0.0173 (8)	0.0176 (8)	0.0153 (7)
C8	0.0445 (10)	0.0427 (9)	0.0312 (8)	0.0203 (8)	0.0099 (7)	0.0182 (7)
C9	0.0377 (8)	0.0313 (7)	0.0243 (7)	0.0147 (6)	0.0103 (6)	0.0098 (6)
C10	0.0358 (9)	0.0340 (8)	0.0330 (8)	0.0080 (7)	0.0057 (7)	0.0102 (6)
C11	0.0452 (10)	0.0275 (7)	0.0399 (9)	0.0103 (7)	0.0131 (8)	0.0125 (7)
C12	0.0421 (9)	0.0297 (7)	0.0338 (8)	0.0179 (7)	0.0118 (7)	0.0134 (6)
C13	0.0368 (8)	0.0278 (7)	0.0258 (7)	0.0158 (6)	0.0105 (6)	0.0079 (5)
C14	0.0388 (9)	0.0341 (8)	0.0302 (8)	0.0141 (7)	0.0078 (7)	0.0088 (6)
C15	0.0272 (8)	0.0317 (7)	0.0355 (8)	0.0074 (6)	0.0078 (6)	0.0098 (6)
C16	0.0402 (10)	0.0376 (9)	0.0483 (10)	0.0147 (7)	0.0123 (8)	0.0139 (8)
C17	0.0500 (12)	0.0358 (9)	0.0604 (13)	0.0164 (8)	0.0178 (10)	0.0059 (8)
C18	0.0597 (13)	0.0473 (11)	0.0450 (11)	0.0165 (10)	0.0130 (10)	−0.0011 (9)
C19	0.0531 (12)	0.0484 (11)	0.0329 (9)	0.0151 (9)	0.0063 (8)	0.0039 (8)
C20	0.0327 (8)	0.0362 (8)	0.0331 (8)	0.0085 (7)	0.0068 (7)	0.0081 (7)
C21	0.0269 (8)	0.0380 (8)	0.0327 (8)	0.0073 (6)	0.0045 (6)	0.0110 (7)
C22	0.0256 (7)	0.0339 (8)	0.0339 (8)	0.0097 (6)	0.0075 (6)	0.0120 (6)
C23	0.0327 (9)	0.0425 (9)	0.0452 (10)	0.0172 (7)	0.0105 (8)	0.0173 (8)
C24	0.0392 (10)	0.0427 (9)	0.0541 (11)	0.0202 (8)	0.0176 (9)	0.0124 (8)
C25	0.0465 (11)	0.0472 (10)	0.0386 (9)	0.0188 (8)	0.0157 (8)	0.0059 (8)
C26	0.0394 (9)	0.0441 (9)	0.0321 (8)	0.0153 (8)	0.0097 (7)	0.0107 (7)
C27	0.0251 (7)	0.0333 (7)	0.0329 (8)	0.0081 (6)	0.0083 (6)	0.0103 (6)
C28	0.0247 (8)	0.0403 (8)	0.0370 (8)	0.0071 (6)	0.0103 (7)	0.0168 (7)
C29	0.0315 (9)	0.0562 (11)	0.0479 (10)	0.0122 (8)	0.0106 (8)	0.0303 (9)
C30	0.0346 (10)	0.0521 (11)	0.0726 (14)	0.0173 (8)	0.0172 (10)	0.0382 (11)
C31	0.0388 (10)	0.0374 (9)	0.0721 (14)	0.0165 (8)	0.0181 (10)	0.0214 (9)
C32	0.0366 (9)	0.0340 (8)	0.0459 (10)	0.0139 (7)	0.0129 (8)	0.0119 (7)
C33	0.0274 (8)	0.0309 (7)	0.0359 (8)	0.0089 (6)	0.0112 (6)	0.0126 (6)
C34	0.0276 (7)	0.0285 (7)	0.0297 (7)	0.0082 (6)	0.0105 (6)	0.0076 (6)
C35	0.0304 (8)	0.0289 (7)	0.0313 (8)	0.0085 (6)	0.0122 (6)	0.0077 (6)
C36	0.0413 (9)	0.0339 (8)	0.0383 (9)	0.0145 (7)	0.0157 (8)	0.0110 (7)
C37	0.0541 (12)	0.0359 (9)	0.0586 (12)	0.0215 (8)	0.0256 (10)	0.0137 (8)
C38	0.0686 (14)	0.0380 (9)	0.0547 (12)	0.0198 (9)	0.0348 (11)	0.0055 (8)
C39	0.0558 (12)	0.0419 (9)	0.0355 (9)	0.0106 (8)	0.0217 (9)	0.0033 (7)
C40	0.0319 (8)	0.0351 (8)	0.0319 (8)	0.0062 (6)	0.0128 (7)	0.0072 (6)
N1	0.0453 (8)	0.0257 (6)	0.0286 (6)	0.0130 (6)	0.0116 (6)	0.0098 (5)
N2	0.0364 (7)	0.0258 (6)	0.0236 (6)	0.0139 (5)	0.0095 (5)	0.0069 (5)
N3	0.0337 (7)	0.0358 (7)	0.0306 (7)	0.0125 (6)	0.0070 (6)	0.0125 (6)
N4	0.0556 (11)	0.0490 (10)	0.0319 (8)	0.0210 (8)	0.0027 (7)	0.0129 (7)
N5	0.0361 (8)	0.0462 (8)	0.0268 (7)	0.0098 (6)	0.0092 (6)	0.0106 (6)
N6	0.0538 (10)	0.0346 (7)	0.0295 (7)	0.0224 (7)	0.0134 (7)	0.0085 (6)
O1	0.0649 (9)	0.0268 (5)	0.0394 (7)	0.0151 (6)	0.0258 (6)	0.0101 (5)
O2	0.0661 (9)	0.0417 (7)	0.0405 (7)	0.0155 (6)	0.0283 (7)	0.0026 (6)
O3	0.0672 (9)	0.0363 (6)	0.0461 (7)	0.0177 (6)	0.0302 (7)	0.0097 (6)
O4	0.0685 (10)	0.0577 (9)	0.0557 (9)	0.0193 (8)	0.0371 (8)	0.0230 (7)
O5	0.0600 (9)	0.0390 (7)	0.0414 (7)	0.0248 (6)	0.0132 (6)	0.0202 (5)
O6	0.0558 (9)	0.0604 (9)	0.0535 (9)	0.0176 (7)	−0.0084 (7)	0.0267 (7)
O7	0.0516 (8)	0.0318 (6)	0.0496 (8)	0.0099 (6)	−0.0026 (7)	0.0055 (6)
O8	0.0411 (7)	0.0431 (7)	0.0471 (7)	0.0201 (6)	0.0009 (6)	0.0107 (6)
O1W	0.0835 (13)	0.0531 (10)	0.0467 (9)	0.0119 (9)	0.0106 (9)	0.0166 (8)
O2W	0.0492 (10)	0.0411 (8)	0.0809 (12)	0.0085 (7)	0.0180 (9)	0.0082 (8)
O3W	0.0691 (12)	0.0796 (12)	0.0318 (8)	−0.0060 (10)	0.0135 (8)	0.0114 (8)
Cu1	0.04830 (13)	0.02337 (9)	0.02931 (10)	0.01400 (8)	0.01495 (9)	0.00781 (7)

### Geometric parameters (Å, °)

**Table:**

C1—O2	1.238 (2)	C24—H24	0.9300
C1—O1	1.264 (2)	C25—C26	1.356 (3)
C1—C2	1.515 (2)	C25—H25	0.9300
C2—N1	1.327 (2)	C26—C27	1.409 (2)
C2—C3	1.382 (2)	C26—H26	0.9300
C3—C4	1.385 (3)	C27—N3	1.355 (2)
C3—H3	0.9300	C28—N5	1.361 (2)
C4—C5	1.384 (3)	C28—C29	1.405 (3)
C4—H4	0.9300	C28—C33	1.408 (2)
C5—C6	1.386 (3)	C29—C30	1.361 (3)
C5—H5	0.9300	C29—H29	0.9300
C6—N1	1.334 (2)	C30—C31	1.396 (3)
C6—C7	1.519 (3)	C30—H30	0.9300
C7—O4	1.231 (2)	C31—C32	1.370 (3)
C7—O3	1.275 (2)	C31—H31	0.9300
C8—O6	1.242 (2)	C32—C33	1.412 (2)
C8—O5	1.255 (2)	C32—H32	0.9300
C8—C9	1.522 (2)	C33—C34	1.437 (2)
C9—N2	1.334 (2)	C34—N6	1.312 (2)
C9—C10	1.386 (2)	C34—C35	1.439 (2)
C10—C11	1.384 (3)	C35—C40	1.405 (2)
C10—H10	0.9300	C35—C36	1.411 (2)
C11—C12	1.378 (3)	C36—C37	1.365 (2)
C11—H11	0.9300	C36—H36	0.9300
C12—C13	1.386 (2)	C37—C38	1.396 (3)
C12—H12	0.9300	C37—H37	0.9300
C13—N2	1.344 (2)	C38—C39	1.357 (3)
C13—C14	1.518 (3)	C38—H38	0.9300
C14—O7	1.243 (2)	C39—C40	1.413 (2)
C14—O8	1.256 (2)	C39—H39	0.9300
C15—N3	1.355 (2)	C40—N5	1.353 (2)
C15—C20	1.410 (2)	N1—Cu1	1.9128 (15)
C15—C16	1.411 (2)	N2—Cu1	1.9818 (14)
C16—C17	1.367 (3)	N3—H3B	0.83 (2)
C16—H16	0.9300	N4—H4B	0.89 (3)
C17—C18	1.395 (3)	N4—H4C	0.78 (3)
C17—H17	0.9300	N5—H5B	0.80 (2)
C18—C19	1.361 (3)	N6—H6A	0.87 (2)
C18—H18	0.9300	N6—H6B	0.83 (3)
C19—C20	1.415 (3)	O1—Cu1	2.0680 (14)
C19—H19	0.9300	O3—Cu1	2.0529 (15)
C20—C21	1.434 (2)	O5—Cu1	2.3150 (18)
C21—N4	1.324 (2)	O7—Cu1	2.3509 (16)
C21—C22	1.430 (2)	O1W—H1WA	0.87 (3)
C22—C27	1.412 (2)	O1W—H1WB	0.74 (3)
C22—C23	1.422 (2)	O2W—H2WA	0.85 (3)
C23—C24	1.358 (3)	O2W—H2WB	0.84 (3)
C23—H23	0.9300	O3W—H3WA	0.79 (3)
C24—C25	1.407 (3)	O3W—H3WB	0.84 (3)
			
O2—C1—O1	125.65 (17)	C26—C27—C22	120.30 (16)
O2—C1—C2	119.24 (15)	N5—C28—C29	119.02 (17)
O1—C1—C2	115.11 (15)	N5—C28—C33	120.47 (16)
N1—C2—C3	120.24 (17)	C29—C28—C33	120.51 (17)
N1—C2—C1	112.35 (14)	C30—C29—C28	119.69 (19)
C3—C2—C1	127.41 (16)	C30—C29—H29	120.2
C2—C3—C4	118.10 (18)	C28—C29—H29	120.2
C2—C3—H3	120.9	C29—C30—C31	120.72 (18)
C4—C3—H3	120.9	C29—C30—H30	119.6
C5—C4—C3	120.66 (17)	C31—C30—H30	119.6
C5—C4—H4	119.7	C32—C31—C30	120.51 (19)
C3—C4—H4	119.7	C32—C31—H31	119.7
C4—C5—C6	118.41 (18)	C30—C31—H31	119.7
C4—C5—H5	120.8	C31—C32—C33	120.44 (19)
C6—C5—H5	120.8	C31—C32—H32	119.8
N1—C6—C5	119.60 (17)	C33—C32—H32	119.8
N1—C6—C7	112.28 (15)	C28—C33—C32	118.12 (16)
C5—C6—C7	128.11 (17)	C28—C33—C34	118.97 (15)
O4—C7—O3	126.43 (18)	C32—C33—C34	122.91 (16)
O4—C7—C6	119.43 (17)	N6—C34—C33	122.09 (15)
O3—C7—C6	114.13 (16)	N6—C34—C35	119.76 (15)
O6—C8—O5	128.25 (17)	C33—C34—C35	118.14 (14)
O6—C8—C9	115.80 (16)	C40—C35—C36	118.52 (15)
O5—C8—C9	115.94 (16)	C40—C35—C34	119.16 (15)
N2—C9—C10	121.76 (15)	C36—C35—C34	122.21 (15)
N2—C9—C8	116.30 (14)	C37—C36—C35	120.43 (18)
C10—C9—C8	121.93 (16)	C37—C36—H36	119.8
C11—C10—C9	118.74 (16)	C35—C36—H36	119.8
C11—C10—H10	120.6	C36—C37—C38	120.26 (19)
C9—C10—H10	120.6	C36—C37—H37	119.9
C12—C11—C10	119.29 (16)	C38—C37—H37	119.9
C12—C11—H11	120.4	C39—C38—C37	121.25 (18)
C10—C11—H11	120.4	C39—C38—H38	119.4
C11—C12—C13	119.24 (16)	C37—C38—H38	119.4
C11—C12—H12	120.4	C38—C39—C40	119.34 (18)
C13—C12—H12	120.4	C38—C39—H39	120.3
N2—C13—C12	121.16 (15)	C40—C39—H39	120.3
N2—C13—C14	115.54 (14)	N5—C40—C35	120.55 (15)
C12—C13—C14	123.27 (15)	N5—C40—C39	119.33 (17)
O7—C14—O8	125.93 (17)	C35—C40—C39	120.11 (17)
O7—C14—C13	116.55 (16)	C2—N1—C6	122.94 (15)
O8—C14—C13	117.52 (15)	C2—N1—Cu1	118.54 (12)
N3—C15—C20	120.58 (16)	C6—N1—Cu1	118.50 (12)
N3—C15—C16	119.34 (17)	C9—N2—C13	119.80 (14)
C20—C15—C16	120.07 (17)	C9—N2—Cu1	119.28 (11)
C17—C16—C15	119.72 (19)	C13—N2—Cu1	120.54 (11)
C17—C16—H16	120.1	C27—N3—C15	122.43 (15)
C15—C16—H16	120.1	C27—N3—H3B	118.2 (15)
C16—C17—C18	120.81 (19)	C15—N3—H3B	119.2 (15)
C16—C17—H17	119.6	C21—N4—H4B	121.0 (16)
C18—C17—H17	119.6	C21—N4—H4C	119 (2)
C19—C18—C17	120.29 (19)	H4B—N4—H4C	119 (3)
C19—C18—H18	119.9	C40—N5—C28	122.49 (15)
C17—C18—H18	119.9	C40—N5—H5B	118.6 (17)
C18—C19—C20	121.1 (2)	C28—N5—H5B	117.4 (17)
C18—C19—H19	119.4	C34—N6—H6A	122.4 (15)
C20—C19—H19	119.4	C34—N6—H6B	120.2 (17)
C15—C20—C19	117.99 (17)	H6A—N6—H6B	117 (2)
C15—C20—C21	118.98 (16)	C1—O1—Cu1	114.16 (11)
C19—C20—C21	122.98 (17)	C7—O3—Cu1	115.04 (12)
N4—C21—C22	120.31 (17)	C8—O5—Cu1	111.55 (11)
N4—C21—C20	121.26 (17)	C14—O7—Cu1	111.16 (12)
C22—C21—C20	118.43 (15)	H1WA—O1W—H1WB	99 (3)
C27—C22—C23	117.95 (16)	H2WA—O2W—H2WB	105 (3)
C27—C22—C21	119.13 (15)	H3WA—O3W—H3WB	110 (3)
C23—C22—C21	122.92 (16)	N1—Cu1—N2	174.31 (6)
C24—C23—C22	120.64 (17)	N1—Cu1—O3	79.84 (6)
C24—C23—H23	119.7	N2—Cu1—O3	104.50 (6)
C22—C23—H23	119.7	N1—Cu1—O1	79.84 (6)
C23—C24—C25	120.51 (18)	N2—Cu1—O1	95.97 (6)
C23—C24—H24	119.7	O3—Cu1—O1	159.47 (5)
C25—C24—H24	119.7	N1—Cu1—O5	107.30 (7)
C26—C25—C24	120.74 (18)	N2—Cu1—O5	76.29 (6)
C26—C25—H25	119.6	O3—Cu1—O5	94.39 (6)
C24—C25—H25	119.6	O1—Cu1—O5	88.91 (6)
C25—C26—C27	119.86 (18)	N1—Cu1—O7	101.06 (7)
C25—C26—H26	120.1	N2—Cu1—O7	75.34 (6)
C27—C26—H26	120.1	O3—Cu1—O7	91.72 (6)
N3—C27—C26	119.27 (16)	O1—Cu1—O7	94.95 (6)
N3—C27—C22	120.43 (15)	O5—Cu1—O7	151.61 (5)
			
O2—C1—C2—N1	178.92 (17)	C34—C35—C36—C37	177.81 (17)
O1—C1—C2—N1	−0.9 (2)	C35—C36—C37—C38	1.0 (3)
O2—C1—C2—C3	−1.3 (3)	C36—C37—C38—C39	−2.0 (3)
O1—C1—C2—C3	178.93 (18)	C37—C38—C39—C40	0.3 (3)
N1—C2—C3—C4	1.4 (3)	C36—C35—C40—N5	175.36 (16)
C1—C2—C3—C4	−178.39 (19)	C34—C35—C40—N5	−0.9 (2)
C2—C3—C4—C5	−0.3 (3)	C36—C35—C40—C39	−3.3 (2)
C3—C4—C5—C6	−1.4 (3)	C34—C35—C40—C39	−179.61 (16)
C4—C5—C6—N1	2.2 (3)	C38—C39—C40—N5	−176.33 (18)
C4—C5—C6—C7	−176.8 (2)	C38—C39—C40—C35	2.4 (3)
N1—C6—C7—O4	−178.97 (18)	C3—C2—N1—C6	−0.7 (3)
C5—C6—C7—O4	0.0 (3)	C1—C2—N1—C6	179.14 (16)
N1—C6—C7—O3	0.0 (2)	C3—C2—N1—Cu1	−179.19 (14)
C5—C6—C7—O3	179.0 (2)	C1—C2—N1—Cu1	0.6 (2)
O6—C8—C9—N2	−171.91 (16)	C5—C6—N1—C2	−1.1 (3)
O5—C8—C9—N2	6.9 (2)	C7—C6—N1—C2	177.94 (16)
O6—C8—C9—C10	7.0 (3)	C5—C6—N1—Cu1	177.36 (15)
O5—C8—C9—C10	−174.13 (16)	C7—C6—N1—Cu1	−3.6 (2)
N2—C9—C10—C11	0.4 (3)	C10—C9—N2—C13	−1.5 (2)
C8—C9—C10—C11	−178.49 (16)	C8—C9—N2—C13	177.47 (14)
C9—C10—C11—C12	0.9 (3)	C10—C9—N2—Cu1	171.40 (13)
C10—C11—C12—C13	−1.1 (3)	C8—C9—N2—Cu1	−9.66 (18)
C11—C12—C13—N2	0.1 (2)	C12—C13—N2—C9	1.2 (2)
C11—C12—C13—C14	−177.93 (16)	C14—C13—N2—C9	179.38 (13)
N2—C13—C14—O7	−10.9 (2)	C12—C13—N2—Cu1	−171.55 (12)
C12—C13—C14—O7	167.20 (17)	C14—C13—N2—Cu1	6.61 (18)
N2—C13—C14—O8	168.95 (15)	C26—C27—N3—C15	−179.52 (16)
C12—C13—C14—O8	−12.9 (2)	C22—C27—N3—C15	0.4 (2)
N3—C15—C16—C17	178.92 (17)	C20—C15—N3—C27	0.5 (2)
C20—C15—C16—C17	−0.7 (3)	C16—C15—N3—C27	−179.13 (16)
C15—C16—C17—C18	0.2 (3)	C35—C40—N5—C28	−3.1 (3)
C16—C17—C18—C19	0.3 (4)	C39—C40—N5—C28	175.61 (16)
C17—C18—C19—C20	−0.4 (3)	C29—C28—N5—C40	−176.57 (16)
N3—C15—C20—C19	−178.98 (17)	C33—C28—N5—C40	3.4 (2)
C16—C15—C20—C19	0.6 (3)	O2—C1—O1—Cu1	−179.08 (16)
N3—C15—C20—C21	−1.6 (2)	C2—C1—O1—Cu1	0.7 (2)
C16—C15—C20—C21	178.05 (16)	O4—C7—O3—Cu1	−177.91 (17)
C18—C19—C20—C15	−0.1 (3)	C6—C7—O3—Cu1	3.2 (2)
C18—C19—C20—C21	−177.4 (2)	O6—C8—O5—Cu1	177.28 (18)
C15—C20—C21—N4	−177.40 (17)	C9—C8—O5—Cu1	−1.39 (19)
C19—C20—C21—N4	−0.1 (3)	O8—C14—O7—Cu1	−170.74 (15)
C15—C20—C21—C22	1.7 (2)	C13—C14—O7—Cu1	9.11 (19)
C19—C20—C21—C22	178.99 (17)	C2—N1—Cu1—O3	−177.34 (14)
N4—C21—C22—C27	178.29 (17)	C6—N1—Cu1—O3	4.08 (13)
C20—C21—C22—C27	−0.9 (2)	C2—N1—Cu1—O1	−0.23 (13)
N4—C21—C22—C23	−1.6 (3)	C6—N1—Cu1—O1	−178.81 (14)
C20—C21—C22—C23	179.26 (16)	C2—N1—Cu1—O5	−85.87 (14)
C27—C22—C23—C24	−0.4 (3)	C6—N1—Cu1—O5	95.56 (14)
C21—C22—C23—C24	179.45 (17)	C2—N1—Cu1—O7	92.88 (14)
C22—C23—C24—C25	1.3 (3)	C6—N1—Cu1—O7	−85.69 (14)
C23—C24—C25—C26	−1.3 (3)	C9—N2—Cu1—O3	97.70 (12)
C24—C25—C26—C27	0.5 (3)	C13—N2—Cu1—O3	−89.48 (13)
C25—C26—C27—N3	−179.69 (17)	C9—N2—Cu1—O1	−80.76 (12)
C25—C26—C27—C22	0.4 (3)	C13—N2—Cu1—O1	92.06 (12)
C23—C22—C27—N3	179.67 (15)	C9—N2—Cu1—O5	6.65 (11)
C21—C22—C27—N3	−0.2 (2)	C13—N2—Cu1—O5	179.47 (13)
C23—C22—C27—C26	−0.4 (2)	C9—N2—Cu1—O7	−174.33 (13)
C21—C22—C27—C26	179.73 (16)	C13—N2—Cu1—O7	−1.52 (12)
N5—C28—C29—C30	179.39 (17)	C7—O3—Cu1—N1	−3.98 (14)
C33—C28—C29—C30	−0.6 (3)	C7—O3—Cu1—N2	172.25 (14)
C28—C29—C30—C31	1.1 (3)	C7—O3—Cu1—O1	−12.1 (3)
C29—C30—C31—C32	−0.5 (3)	C7—O3—Cu1—O5	−110.80 (15)
C30—C31—C32—C33	−0.7 (3)	C7—O3—Cu1—O7	96.94 (15)
N5—C28—C33—C32	179.48 (15)	C1—O1—Cu1—N1	−0.30 (13)
C29—C28—C33—C32	−0.6 (2)	C1—O1—Cu1—N2	−176.41 (13)
N5—C28—C33—C34	0.4 (2)	C1—O1—Cu1—O3	7.8 (3)
C29—C28—C33—C34	−179.68 (15)	C1—O1—Cu1—O5	107.50 (14)
C31—C32—C33—C28	1.2 (3)	C1—O1—Cu1—O7	−100.67 (14)
C31—C32—C33—C34	−179.76 (17)	C8—O5—Cu1—N1	172.87 (12)
C28—C33—C34—N6	177.13 (16)	C8—O5—Cu1—N2	−2.54 (12)
C32—C33—C34—N6	−1.9 (3)	C8—O5—Cu1—O3	−106.42 (13)
C28—C33—C34—C35	−4.1 (2)	C8—O5—Cu1—O1	93.87 (13)
C32—C33—C34—C35	176.79 (15)	C8—O5—Cu1—O7	−4.55 (19)
N6—C34—C35—C40	−176.82 (16)	C14—O7—Cu1—N1	179.90 (13)
C33—C34—C35—C40	4.4 (2)	C14—O7—Cu1—N2	−4.63 (13)
N6—C34—C35—C36	7.1 (2)	C14—O7—Cu1—O3	99.91 (14)
C33—C34—C35—C36	−171.71 (15)	C14—O7—Cu1—O1	−99.52 (14)
C40—C35—C36—C37	1.7 (3)	C14—O7—Cu1—O5	−2.6 (2)

### Hydrogen-bond geometry (Å, °)

**Table:**

*D*—H···*A*	*D*—H	H···*A*	*D*···*A*	*D*—H···*A*
O1W—H1WA···O7	0.87 (4)	1.98 (4)	2.832 (2)	167 (4)
O1W—H1WB···O4^i^	0.74 (4)	2.14 (4)	2.877 (3)	175 (4)
O2W—H2WA···O8^ii^	0.84 (3)	1.98 (3)	2.820 (3)	174 (3)
O2W—H2WB···O8	0.84 (4)	1.98 (4)	2.810 (3)	174 (4)
O3W—H3WA···O2^iii^	0.79 (3)	2.01 (3)	2.787 (3)	169 (3)
O3W—H3WB···O2W^iv^	0.84 (3)	1.89 (3)	2.730 (3)	175 (3)
N3—H3B···O6	0.83 (2)	1.88 (2)	2.716 (2)	179 (3)
N4—H4B···O1W^v^	0.89 (3)	2.09 (3)	2.938 (3)	158 (2)
N4—H4C···O8^v^	0.77 (3)	2.27 (3)	2.972 (2)	152 (3)
N5—H5B···O3W	0.80 (2)	1.91 (2)	2.703 (2)	173 (2)
N6—H6A···O2^vi^	0.87 (2)	1.97 (2)	2.819 (2)	164 (2)
N6—H6B···O5	0.83 (3)	2.11 (3)	2.888 (2)	158 (2)

Symmetry codes: (i) −*x*+1, −*y*+1, −*z*+1; (ii) −*x*+1, −*y*+2, −*z*+1; (iii) *x*, *y*, *z*+1; (iv) −*x*+2, −*y*+2, −*z*+2; (v) *x*+1, *y*, *z*+1; (vi) −*x*+2, −*y*+1, −*z*+1.
